# Improvement of NMDA encephalitis by active lymph node removal

**DOI:** 10.1007/s00540-013-1759-5

**Published:** 2013-12-20

**Authors:** Tatsushige Iwamoto, Yasuhiro Shiokawa, Shinichi Nakao

**Affiliations:** Department of Anesthesiology, Kinki University Faculty of Medicine, 377-2 Ohno-higashi, Osakasayama City, Osaka 589-8511 Japan

**Keywords:** Anti-NMDAR encephalitis, Image diagnosis

To the Editor:

Anti-*N*-methyl-*D*-aspartate-receptor (NMDAR) encephalitis is caused by serum antibodies against NMDAR, and is often associated with tumors [1]. Its symptoms include psychosis, seizures and disturbance of consciousness, all of which resemble the symptoms induced by NMDA antagonists, such as ketamine. We report a patient with anti-NMDAR encephalitis, and we had difficulty treating the patient because no primary tumor was found.

A 57-year-old woman, who had a cough and fever, developed to status epilepticus, and consciousness disturbance. Examinations revealed elevated anti-NMDAR antibodies, but no obvious primary tumor could be found with a CT scan. However, fluorodeoxyglucose positron emission tomography showed an accumulation of fluorescence in a right enlarged obturator lymph node (Fig. 1, Supplementary material), indicating augmented metabolism, and lymph node removal was scheduled. After epidural catheter placement, anesthesia was induced with propofol, and the trachea was intubated. Anesthesia was maintained with 60 % nitrous oxide and 1.5 % sevoflurane with epidural anesthesia. The surgery was completed without complications. After tracheal extubation, the patient was transferred to the intensive care unit. The patient’s postoperative course was uneventful. Her lymph node was histopathologically normal, but we postulated that the lymph node would produce ectopic NMDAR. The patient’s level of consciousness improved gradually.

There are some anesthetic drugs that serve as NMDAR antagonists, such as ketamine and nitrous oxide. Pryzbylkowski et al. [2] have recommended avoiding such drugs for anesthesia in a patient with anti-NMDAR encephalitis, and we should not have used nitrous oxide for anesthesia.

In conclusion, we encountered a patient with anti-NMDAR encephalitis without a responsible tumor, but removal of a metabolically active enlarged lymph node markedly improved the symptoms.

## Electronic supplementary material

Below is the link to the electronic supplementary material.

(PPTX 636 kb)
