# Supplementary material for: Improvement of NMDA encephalitis by active lymph node removal
Source: J Anesth. 2013 Dec 20;28(4):646. doi: 10.1007/s00540-013-1759-5 (PMC4126996; doi:10.1007/s00540-013-1759-5)
Supplement: Supplementary file 1 — (PPTX 636 kb) [file 540_2013_1759_MOESM1_ESM.pptx]

## Slide 1
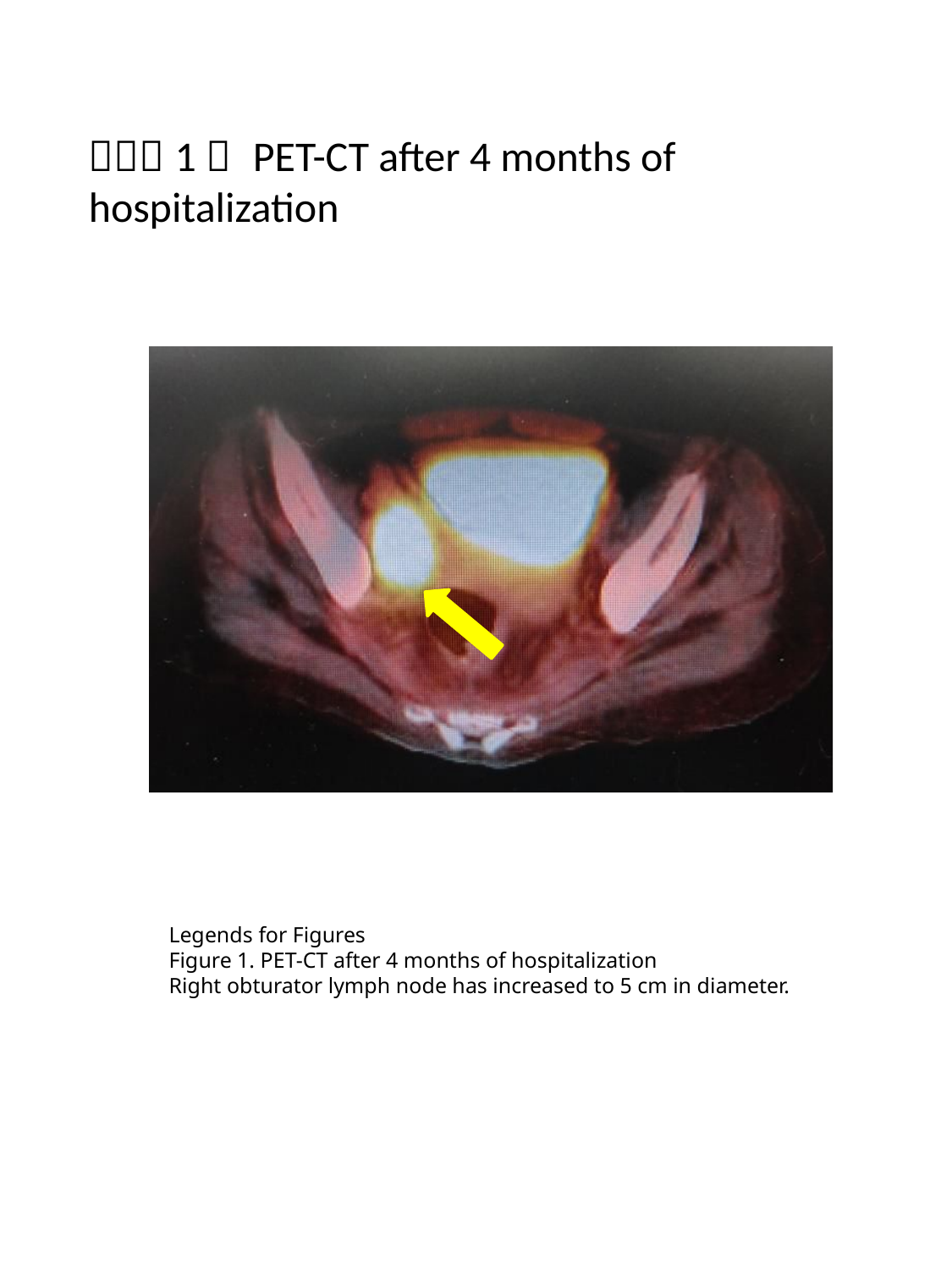

Ｆｉｇ1： PET-CT after 4 months of hospitalization
Legends for Figures
Figure 1. PET-CT after 4 months of hospitalization
Right obturator lymph node has increased to 5 cm in diameter.
